# Psychiatric Emergency or General Emergency: Evolution or Involution? A Qualitative Study With Mental Health and Emergency Professionals

**DOI:** 10.1111/inm.70063

**Published:** 2025-05-19

**Authors:** Camuccio Carlo Alberto, Zara Silvia

**Affiliations:** ^1^ Mental Health Nursing, Dimed University of Padua Padua Italy; ^2^ Immunology and Molecular Oncology Diagnostics Unit Veneto Institute of Oncology IOV‐IRCCS Padua Italy; ^3^ Maternal and Child Department Hospital Treviso, Aulss 2 Marca Trevigiana Treviso Italy

**Keywords:** emergency treatment, mental health, nurses, prejudice, psychiatry, stigma

## Abstract

The treatment of individuals with psychiatric disorders who visit the Emergency Department (ED) remains a significant issue within healthcare organisations. Over the past decades, various organisational solutions have been proposed, ranging from dedicated Emergency Departments to liaison mechanisms involving mental health nurses within EDs or direct access to acute units. On one hand, there are clinical and organisational needs pushing towards the creation of dedicated pathways; on the other hand, there are concerns that such solutions may be counterproductive and dangerous in terms of health and social inclusion. The aim of this study is to assess the opinions of Mental Health and Emergency professionals on the advantages and disadvantages of clinical and organisational pathways dedicated to patients with psychiatric disorders who visit the general ED. The study was conducted using a qualitative research approach: semi‐structured interviews were carried out through purposeful sampling composed of two cohorts: Emergency and Mental Health professionals. The data were analysed using content analysis with the software Atlas.ti. Forty‐five interviews were collected, and six main themes/families were identified. A certain distance in opinions between the two cohorts emerged, especially regarding the adoption of dedicated pathways. In both cohorts, but particularly in the mental health cohort, there is a fear of stigmatisation and violation of patients' rights in dedicated pathways. Both groups believe that there is a need for more specific training and greater multidisciplinarity. This study adheres to the COREQ checklist for qualitative studies.

## 1 Introduction

In the literature, psychiatric emergencies are the third most common, following those in internal medicine and surgery (Pajonk et al. 2001). Mental health issues account for nearly 10% and 12% of emergency department (ED) visits according to national data from Canada and the United States, respectively (Navas et al. 2022). These patients utilise emergency services at significantly higher rates compared to the general population; when considering the presence of a psychiatric disorder in conjunction with other diagnoses, the total prevalence of people with psychiatric disorders in ED is 38%, as confirmed by Fleury et al. (2024).

However, the high utilisation of emergency services occurs in patients who frequently have medical conditions alongside psychiatric disorders: indeed, Tucci et al. (2017) reported that 34%–50% of psychiatric patients in ED have coexisting medical diseases that can exacerbate their psychiatric symptomatology. Moreover, the meta‐analysis of Firth et al. (2019) shows how people with mental health diagnoses have from 1.4 to 2 increased risk of cardiovascular diseases. So, we can conclude that this typology of patients frequently presents medical conditions that lead to a significantly reduced life expectancy compared to the general population (Hardy et al. 2011).

These patients often express their physical discomfort not clearly but rather confusingly, frequently presenting an exacerbation of psychiatric symptoms that can mask the physical ones. This makes it difficult for non‐mental health staff to distinguish between psychiatric and medical conditions. Consequently, the lack of training and aptitude in mental health leads to frequent underestimation of physical symptoms in favour of psychiatric ones, resulting in missed diagnoses, the phenomenon of diagnostic overshadowing, and premature discharges (Gillard et al. 2023; Firth et al. 2019). In light of these data, a proposed and adopted solution in many countries is the establishment of ED and/or dedicated facilities and pathways for patients with mental disorders. Pathways that emerge, thanks to the presence of mental health experts, provide a better and timely response to psychiatric needs, while also allowing for a more accurate assessment of physical symptoms, distinguishing them from psychiatric ones (Postorivo et al. 2024). Many healthcare professionals and administrators consider dedicated pathways valid solutions for both patients and hospital organisations, taking into account issues such as the increasing demand for healthcare, which manifests in the chronic phenomenon of ED overcrowding.

The literature shows numerous alternative solutions, describing operational experiences that range from Psychiatric Emergency to liaison services and fast‐track interventions, with several other organisational types in between (Postorivo et al. 2024; Anderson et al. 2022; Schmidt et al. 2020; Slade et al. 2007). Moreover, in many EDs, the role of the mental health nurse has been introduced, acting as a ‘gatekeeper’ or managing psychiatric emergencies (Wand et al. 2011a, 2011b; Nicholls et al. 2010).

### 1.1 Background

According to the authors, these solutions have benefits for patients as they reduce waiting times, preserve privacy and offer direct access to specialised mental health staff (Frank et al. 2005).

But, some, particularly mental health professionals, criticise these solutions, arguing that such services are; dangerous and discriminatory,’ introducing numerous clinical risks to patients and contributing to the stigma of people with mental disorders. Moreover for Harris et al. (2016), there is a ‘lack of care’ in terms of specific infrastructure, staff competencies and available materials and resources. This situation has consequences for patients, as diagnostic overshadowing (Hallyburton 2022) and for staff, who are often unprepared to manage these cases.

But in general, ED institutional, physical and environmental barriers, along with a lack of knowledge, slow down the process of properly managing patients with psychiatric conditions. It is noted that psychiatric patients in acute phases perceive certain characteristics of the ED (such as lights, noise and confusion) as more intense, disturbing and difficult to handle compared to other users without psychiatric conditions. This environmental aspect is compounded by the human aspect, where patients feel a lack of understanding and support from hospital staff (Perrone McIntosh 2020).

However, these advantages could also be seen as a false economy, merely shifting the problem elsewhere. A specialised centre separate from the standard ED might create a separate healthcare system that stigmatises mental health ‘consumers’ (Wand 2005).

In Italy, Psychiatric EDs or clinical organisational solutions that include dedicated structures and pathways are not common. Psychiatric EDs are rare, primarily because psychiatric hospitals do not exist in Italy. Instead, there are community services with acute care units of a maximum of 15 beds, located within general hospitals (Gigantesco et al. 2012).

In the Italian landscape, the most common form of clinical–organisational interaction between ED and psychiatry is psychiatric liaison services or on‐call consultations. More complex forms, with dedicated pathways starting from the community and involving predominantly mental health personnel well‐trained in emergencies, are present but remain minority practices.

The aim of this study is to assess the opinions of Mental Health and Emergency professionals on the advantages and disadvantages of clinical and organisational pathways that are dedicated to patients with psychiatric disorders who visit the general ED. The research questions are: (1) What is the most appropriate treatment modality for patients presenting to the ED with a psychiatric disorder? (2) What are the opinions of ED and MH professionals regarding the creation and development of a Psychiatric Emergency Department or dedicated pathways? (3) What implications would such a creation have for the patient and the fulfilment of their care needs?

## 2 Methods

### 2.1 Design

The study was conducted using a qualitative research approach: semi‐structured interviews were carried out by both the authors with expertise in interviewsthrough purposeful sampling, i.e., with authoritative interviewees (Luciani et al. 2019; Palinkas et al. 2015).

The recruitment process first involved identifying psychiatric and mental health services with a certain influx of patients, typically in cities and urban contexts. We also recruited two services in smaller rural areas. Additionally, we identified two MHDs in two large cities with particular and specific care approaches that are well‐known in the professional community in Italy.

The interviewees included: physicians and nurses from ED and Mental Health Services in three regions of northern Italy. Following a purposive sampling logic, participants were selected based on their ability to provide qualified and authoritative opinions on the phenomenon under study. The medical directors and the nursing manager of the departments (Emergency and MH) provided us with a functional chart to contact professionals, giving us some suggestions. We also included members of the boards of professional national associations for both Emergency‐Urgency and Mental Health: Società Italiana di Medicina d'Emergenza Urgenza—SIMEU (Italian Society of Emergency Medicine) and Società Italiana di Psichiatria sezione Veneto—PSIVE (Italian Society of Psychiatry—Veneto Section).

The sample, of a purposeful nature, was recruited according to the following inclusion criteria:

1. At least 5 years of experience in Emergency–Urgency or Psychiatry/Mental Health.
2. Due to their functional position or experience, they are capable of providing an authoritative opinion on the subject under study.

The exclusion criteria considered were:

1. Newly hired staff.
2. Staff belonging to units other than the ED and Mental Health.
3. Lack of consent to participate in the study.

### 2.2 Data Collection

Data collection was conducted through semi‐structured interviews (Table 1) between early September and mid‐October 2019. Each interview lasted an average of 30–40 min and was preferably conducted face‐to‐face, between the interviewee and the interviewer, but also by telephone. The interviews were audio‐recorded. The question grid was created after a careful review of the literature and by two pilot interviews conducted in the early stages of the project, which were not included in the results. The meetings, both face‐to‐face and by phone, took place in private and quiet environments free from disturbances; the locations where the interviews were held were chosen together with the interviewee.

**TABLE 1:** Interview questions. The table contains the questions that were used as a guide for the semi‐structured interviews conducted in the study.

Interview questions
What is the usual pathway or pathways followed by a person with a Mental Disorder who arrives at the Emergency Department? What do you believe is the most appropriate way to manage a person with a Mental Disorder when they present to the ED with a severe crisis related to their condition? Do you think that a Psychiatric ED with direct access to the ward has positive or negative aspects? If so, what are they? Conversely, what do you believe are the negative aspects associated with the development of such a proposal? Do you believe that admitting a psychiatric patient to a dedicated Psychiatric ED allows for better understanding and treatment of the patient and the crisis related to their condition? Do you think that the Psychiatric ED service could cause stigma in patients with psychiatric conditions? If so, why? Are acute ED or psychiatric staff trained to manage psychiatric patients in emergency?

### 2.3 Data Analysis

The content analysis of the interviews was conducted by the two authors separately, which after numerous readings to familiarise themselves with the material, used the distillation method technique suggested by Downe‐Wamboldt (1992). The two researchers then met to discuss and reconcile any discrepancies in their coding to achieve a shared perspective. The codes and themes that emerged from the two verbatim analyses were verified and compared with the content analysis software Atlas TI version 7 until the creation of definitive thematic families. The field notes filled out at the end of the interview helped the coding process. In both phases, analysed data were reviewed by an independent researcher for verification of themes. Content saturation was constantly monitored.

### 2.4 Ethical Issues

Formal approval of the study was obtained from the Italian Health Authorities and the Local Departments of Mental Health. Confidentiality and anonymity were guaranteed and treated as dictated by Italian law, and the participants were informed that they could withdraw from the study at any time. Before the data were made available to the authors, participants' identifiers were replaced with anonymous codes. To ensure the anonymity of the interviewees, we did not make precise geographical references, nor did we specify formal positions when it comes to members of scientific societies, generally indicating ‘board member’. Given the stringency of this procedure and the nature of the study, Health Authorities considered that it was not necessary to seek authorisation from the local ethics committee. The study was carried out in accordance with the principles of the Declaration of Helsinki. This study adheres to the COREQ checklist for qualitative studies (Tong et al. 2007).

## 3 Results

Content saturation was achieved and collected for both cohorts with 45 interviews: 23 from the Mental Health Area and 22 from the Emergency and Urgency Area (Table 2). Two initial interviews were excluded as they were used as pilot interviews. A response rate of 93% was achieved, given that participation was requested from 48 individuals (Tables 3, 4, 5, 6, 7, 8).

**TABLE 2:** Study sample. This is the description of the interviewed sample divided into the two cohorts of Emergency and Urgent Care and Mental Health. In addition to gender, the table includes the role, years of experience in healthcare, years in the specific area of interest, specific department of affiliation and any special functions.

Code	Sex	Age	Job role	Years of service	Years of service in the specific area	Note
*Mental health*						
1 MH	M	65	Psychiatrist, former Director of the Department of Mental Health	40	40	Former Director of Department
2 MH	F	44	Nurse	23	19	Acute (1,5 in ED)
3 MH	M	38	Clinical nursing ward manager	13	7 as Clinical Ward Manager in acute unit	Acute
4 MH	F	52	Nurse	30	27 years in Psychiatry	Acute
5 MH	F	45	Clinical nursing ward manager	21	19 Clinical Ward Manager	Acute
6 MH	F	29	Nurse	6	6	Acute
8 MH	M	68	Psychiatrist	40	40	Former Department Director; retired
9 I MH	F	44	Clinical nursing ward manager	21	21	Community Mental Health Centre
10 MH	F	42	Psychiatrist	14	10	
11 MH	F	55	Psychiatrist	25	25	
12 MH	F	61	Psychiatrist	28	28	
13 MH	M	50	Clinical nursing ward manager	28	11 as Clinical Ward Manager in acute unit	acute
14 MH	F	46	Clinical nursing ward manager	23	20 Clinical Ward Manager Day Hospital	Day Hospital
15 MH	M	61	Nurse	43	25 in Community Mental Health Centre; 18 in acute unit	Community Mental Health Centre
16 MH	F	56	Department nursing manager	34	31 in Mental Health a Salute Mentale	Mental Health Department
17 MH	M	66	Psychiatrist	35	35	Retired (former Director of Department)
18 MH	M	53	Professor in mental health nursing	32	10 Professor	University
19 MH	M	63	Psychiatrist, professor of Psychiatry	34	34	University
20 MH	F	32	Nurse	8	5 acute unit	Acute
21 MH	F	53	Clinical nursing ward manager	29	8 clinical nursing ward manager acute unit	Acute (restraint and seclusion free)
22 MH	M	53	Psychiatrist	23	17	PSIVE board
23 MH	M	68	Psychiatrist	37	34 (only acute unit)	PSIVE board
*Emegency Department*						
1 ED	F	67	Physician	37	37	ED (retired)
2 ED	M	38	Physician	8	5	ED
3 ED	M	47	Clinical nursing ward manager	25	11 clinical nursing ward manager	ED
4 ED	M	34	Clinical nursing ward manager	10	10 (2 clinical nursing ward manager)	ED
5 ED	M	33	Nurse	8	5 ED	ED (triage instructor)
6 ED	M	34	Nurse	8	5 ED	ED (triage instructor)
8 ED	M	29	Nurse	6	5 ambulance	ED (ambulance; nursing order board)
9 ED	F	55	Clinical nursing ward manager	30	20; 3 as clinical nursing ward manager Inf.	ED
10 ED	F	53	Clinical nursing ward manager	27	21	ED
11 ED	M	35	Nurse	11	9	ED (ambulance; nursing order board)
12 ED	M	38	Nurse	13	13	ED (nurse mentor)
13 ED	F	43	Nurse	18	13	ED
14 ED	M	35	Physician	6	5	ED
15 ED	F	26	Nurse	12	8	ED
16 ED	F	48	Nurse	26	7	ED
17 ED	M	39	Clinical nursing ward manager	13	13	ED
18 ED	F	60	Clinical nursing ward manager	40	5 clinical nursing ward manager	ED
19 ED	F	32	Nurse	7	5	ED
20 ED	M	33	Physician	5	5	ED
21 ED	M	56	Physician	26	23	ED (Department Director; SIMEU board)
22 ED	M	33	Nurse	7	5	ED (SIMEU board)

**TABLE 3:** Families by Atlas TI 7. This table illustrates the six families of codes that the researchers derived using Atlas.ti.

	Famiglie Atlas – Ti 7
Family 1	The usual pathway of the patient with a Psychiatric Disorder
Family 2	Ideal pathway for the patient with Psychiatric Disorder
Family 3	Positive and negative aspects of dedicated tracks
Family 4	Separated accessest: Stigma?
Family 5	ED Staff: trained to manage patients with psychiatric disorders?
Family 6	MH Staff: trained to receive emergency patients?

**TABLE 4:** Codes cohort Family 1. The table for Family 1, usual pathways, shows the codes used and shared by the researchers during the analysis, divided by cohorts.

Mental health	Emergency department
Current mode {13–0} Initial ED assessment {15–0} Psychiatric consultation {10–0} Hospitalisation {5–0} Discharge (community‐referral to home) {6‐0} exclusion of organic causes {6–0} Differential diagnosis {5–0} Standard treatment for psychiatric patients {4–0} Diagnostic tests {4–0} Psychiatrist present 24/7 {3–0} Protected pathway {1–0} Mental health staff consultations in PS {1–0} Professionalism and listening {1–0} Recognition of psychological needs and SS management {1–0}	Current mode {7–0} Initial PS assessment {5–0} Triage {4–0} Fast track {7–0} Exclusion of organic causes {3–0} Early psychiatric consultation {3–0} Psychiatric consultation {4–0} Management of unknown/acute/agitated patients in PS {5–0} Known/non‐critical/non‐agitated patients = direct access {4–0} Create suitable environment for patient {2–0} Listening and understanding {2–0} Anamnesis/differential diagnosis {2–0} Management of community emergencies {2–0}

**TABLE 5:** Codes cohort Family 2. The table for Family 2, Ideal pathways, shows the codes used and shared by the researchers during the analysis, divided by cohorts.

Mental health		Emergency department	
Negative {14–0}	Positive {1–0} e Positive/−Negative {9–0}	Positive/−Negative {12–0}	Positive {7–0} e Positive only for known patients {3–0}
(1) Predetermination of the diagnosis attributed to the patient {9–0}	(1) Reduction of waiting time for Psychiatric Consultation and admission in ED {8–0}	(1) Underestimation of organic internal problems {7–0}	(1) For known, stable, purely psychiatric patients, minor codes, and involuntary treatments {8–0}
(2) Underestimation of organic signs and symptoms {9–0}	(2) Gestione del paziente da parte di personale con competenze specifiche {3–0}	(2) Risk to the safety of healthcare staff/Patients/Patients admitted to the ward; if access of Patients in an agitated state {2–0}	(2) Reduction of waiting time in PS {7–0}
(3) Reduced diagnostic investigations {5–0}	(3) Contained/known environment {3–0}	(3) Risk to the safety of staff {2–0}	(3) Reduction of anxiety and discomfort experienced by the patient {6–0}
(4) Ineffective initial PS assessment {4–0}	(4) More effective crisis management {2–0}		(4) Staff trained with specific skills {6–0}
(5) Improper admissions and hospitalizations {3–0}	(5) Positive if facilitated pathway for patients with Psychiatric Disorders {1–0}		(5) Known environment {5–0}
(6) Insufficient resources available			(6) Reduction of the workload of ED staff
(7) Absence of differential diagnosis {3–0}			(7) Reduction of improper admissions in ED {5–0}
(8) Risk to the safety of the patient/staff {2–0}			
(9) Ineffective emergency management {2–0}			

**TABLE 6:** Codes cohort Family 3. The table for Family 3, Negative and Positive Aspects of Dedicated Tracks, shows the codes used and shared by the researchers during the analysis, divided by cohorts.

Positive and negative aspects of dedicated tracks	Mental health	Emergency department
Yes	{4–0}	{15–0}
No	{14–0}	{3–0}
Yes and No	{5–0}	{4–0}
Advantages	Trained staff Direct care (by the specialist) Reduced waiting time Only for purely psychiatric patients Correct medication administration versus oversedation Direct access for known patients Only if Fast Track (not direct access)	Trained staff with specific skills Suitable/known environment More time for the patient Direct care (by the specialist) Reduced waiting time Immediate treatment Only for purely psychiatric/known patients Correct medication administration Reduced workload for ED Only for purely psychiatric/known patients
Disadvantages	Underestimation of organic SS Diagnostic errors Stigma‐diversity Predetermination of diagnosis Institutionalisation Asylum logic Improper admissions ‘Only lightens the work of ED’ Acute patients (management in ED) Absence of differential diagnosis Reduced quality of care Logistical and organisational problems Threat to the patient's right to health Insufficient staff and resources	Underestimation of organic SS Reduced diagnostic tests Absence of differential diagnosis Risk of ‘labeling’ the patient Acute patients (management in ED) Better Fast Track

**TABLE 7:** Codes cohort Family 4. The table for Family 4 investigates the presence of stigma according to professionals and shows the codes used and shared by the researchers during the analysis, divided by cohorts.

Dedicated accesses: stigma?	Mental health	Emergency department
Yes	{20–0}	{18–0}
No	{3–0}	{4–0}
Yes	Stigma {11–0}; predetermination of diagnosis {8–0}; category and distinction {8–0}; discriminatory signs and symptoms {7–0} (agitated = psychiatric); diagnostic errors {4–0}; ineffective initial assessment {3–0}; asylum logic {2–0}; ‘PSI’ negative connotation {2–0}	Awareness of illness {6–0}, meeting the specialist is the patient's will; Their will {6–0}; pre‐existing stigma {3–0}; in a patient already known/in care of the service, the stigma does not increase {2–0}, with direct access; reduction of stigma; greater privacy for the patient {2–0}
No	Stigma already present, pre‐existing {3–0}	Labeling/marked patient {3–0}; totalitarian structure {1–0}; asylum logic {1–0}; self‐attribution of stigma by the patient {1–0}

**TABLE 8:** Codes cohort Family 5–6. The tables for Families 5 and 6 investigate the training needs of professionals, listing the proposals that emerged and were identified by the researchers during the analysis.

	Mental health	Emergency department
ED Staff: trained to manage patients with psychiatric disorders?		
Yes	{4–0}	{5–0}
NO	{15–0}	{14–0}
Yes and No	{3–0}	{3–0}
Don't know	{1–0}	{0–0}
Suggestions	Mental health training courses {13–0}; relational courses {7–0}; courses for the management of psychiatric emergencies {3–0}; mental health staff consultation in ED {2–0}; multiprofessionalism {2–0}; on‐the‐job training {2–0}; collaboration {3–0}	Advanced psychiatric and mental health training courses {7–0}; creation of protocols for standardised patient management {3–0}; relational and patient approach courses {3–0}; meetings between ED and MH {3–0}; dedicated clinic room in ED {3–0}; mental health staff consultation in ED {2–0}; courses for managing violent‐agitated patients {2–0}
MH Staff: trained to receive emergency patients?		
Yes	{6–0}	{12–0}
NO	{3–0}	{2–0}
Yes and No	{14–0}	{5–0}
Don't know	{0–0}	{3–0}
Suggestions	Integration of PS skills (Interventionist) {5–0}; enhancement of SS management for organic pathologies {6–0}; collaboration {3–0}; relational courses {2–0}; mental health staff consultation in PS Include community nurses in psychiatric PS Promote daytime management of patients with Community staff meetings between PS and SPDC staff On‐the‐job training Define skills/organisation of direct access	Integration of ED skills (critical patient management, speed) {3–0}; emergency management courses {5–0}; reorganisation of acute unit and addition of extra staff {1–0}

Our sample has an average age of 49.7 years for the MH cohort and 39.4 years for the ED cohort. The average years of experience in healthcare are 25.5 for MH and 15.3 for ED, while the average years of experience in the specific sector are 12.3 for MH and 7.6 for ED.

The MH cohort consists of 10 psychiatrists and 13 nurses, of which 7 are nursing managers. Eight nurses work in acute wards, 2 in community mental health centres, one in department management, one in a day hospital, and one at a university as a lecturer. The psychiatrists, unless otherwise specified, work on rotation both in acute settings and in the community. The ED cohort includes 5 doctors and 17 nurses, of which 7 are nursing managers.

Only three people contacted for interviews refused, and despite the different professional categories of each participant, all the themes inherent in the survey were considered important, and all the interviews were of a good level of depth.

### 3.1 Content Analysis

Upon the conclusion of the two described phases of content analysis, various codes were assigned and identified, which were subsequently grouped and organised into specific families of meaning (Table 3). In the current analysis, these families were identified within the six interview questions.

An additional analysis was also conducted using Atlas‐Ti 7, so that each question/family was analysed for each individual cohort under study, namely the Mental Health cohort and the Emergency cohort. This was done to compare the codings and to have a concise comparison tool.

### 3.2 The Usual Pathway of the Patient With a Psychiatric Disorder

The most common approach is a consultation request from the ED to the psychiatry unit, but interviews reveal that in some hospitals there is already a daytime clinic for emergency management with a psychiatrist present 24/7 and not just on call. However, it appears to be a common pathway for patients experiencing psychomotor agitation, especially those suspected of substance abuse, to be initially seen in the ED to prevent any deterioration of their physical condition. Some MH interviewers want to specify that the patient should be treated in the ED but in a dedicated space and not, as often happened in the past, ‘understairs’ (I10 MH).

But according to an Emergency physician:

The patient is seen by the Emergency Department doctor in the clinic, who, if necessary, calls the psychiatrist, who arrives whenever they feel like it. (I1 ED)

Or a nurse who states:

Now the psychiatrist comes to the Emergency Department, and the staff must handle the situation in the meantime. (I12 ED).

But:

The patient with psychiatric disorders is an abstraction. Who is the patient with psychiatric disorders? It depends on the reason for access!… How can one define and evaluate a patient by assigning them a diagnosis just because they are already known to the service, while conducting limited diagnostic tests? (I21 MH).

In the end a former MH Director:

There are broad border areas (psychogeriatrics, cognitive disorders in general, drug addiction, alcoholism) … The comorbidities present in psychiatric disorders should not be underestimated. (I8 MH).

### 3.3 Ideal Pathway for the Patient With Psychiatric Disorder

According to the PSIVE board member, the most appropriate pathway is:

Initial evaluation by the Emergency Department doctor, immediately… blood tests and instrumental exams to be reviewed by the psychiatrist when they arrive for consultation. The psychiatrist is called afterwards, once the patient is stabilized. (I22 MH).

Therefore, a reflection:

With the psychiatric patient, it seems there is an invisible barrier that makes colleagues not want to approach and not consider such patients part of their work. (I22 MH).

Noteworthy, the opinion of all the Mental Health interviewers are in agreement on this approach.

A nurse clinical manager supports an integrated 24‐h community‐acute unit care model presented in her hospital:

We try to implement a process through which the person is guaranteed a rapid evaluation and a treatment and diagnosis framework without too much downtime. (I5MH).

The opinions of emergency professionals tend to differ, proposing differentiated pathways.

For example, according to a nursing member of the SIMEU board.

The current method works, but it might be a good idea to create a clinic in the form of a psychiatric emergency room; from the general ED, I visit and send the patient to the clinic (especially in the case of an unknown patient). For known and non‐critical patients, direct access instead. (I7 ED).

The same view is expressed by a clinical nurse manager of an Emergency:

For us, for all known patients, it would be useful to have a short pathway like a psychiatric fast track to send them directly to psychiatry. It would be better for them because it would reduce the waiting time. (I18 ED).

The distinction between known and unknown patients is also emphasised in terms of pathway and triage by a nurse.

Fast track for known patients, white code; the procedure could be standardized for white codes already followed with known pathology and sent to the psychiatric fast track.(I5 ED).

## 4 There Are Hints of Other Reasons for Not Having Psychiatric Patients Go Through the Emergency Department

From the ambulance's point of view, I would like the psychiatric staff to come to the home… to avoid congestion and overcrowding in the ED (I6 ED).

But all these differentiated pathways, especially for known or unknown patients, or for other reasons, are dismissed by many mental health professionals, as evidenced by these two MH interviewers:

…behind a known patient or agitation, there can be internal and physical issues… (I22 MH).

Every time a seemingly privileged pathway is created for psychiatric patients, it ends up representing an element of stigma and exclusion from rights of care (I8 MH).

### 4.1 Positive and Negative Aspects of Dedicated Tracks

The following family tended to ask about the positive and negative aspects of ED dedicated to psychiatric patients, particularly the fast track directly to acute wards.

A PSIVE board member is very blunt and straightforward, pointing out hidden psychiatrization (Beeker 2022):

I believe this means returning to the concept of the asylum. The asylum is not the walls; it is the separation! Those who want direct access to the ED are asylum advocates and should be labeled as such. The asylum is not the walls; it is the logic that creates an asylum mentality. Those who think this way are territorial asylum advocates, wanting to create asylums in the community. It means you are different. (Int. 7 MH).

The positions of another PSIVE member are more nuanced

Believing that a diagnosis can be made solely based on people's behavioral aspects is a tragic mistake; one must start by ruling out organic pathologies. (Int. 7 MH)

An ethical aspect is echoed in the words of a nurse from an acute unit ‘restraint and seclusion free’.

If I am depressed and having a heart attack, who will treat me? They need to save my life! I do not want the pathways to the right to care for citizens to be excluded. I am convinced that alternative pathways should not be created for what is defined upstream as psychiatric. There are few purely psychiatric patients. (Int. 5 MH).

Emergency professionals appear to be cautiously in favour of solutions oriented towards exclusive pathways and structures for psychiatric patients. This is due to clinical…

Not all psychiatric pathology, as it manifests, can be managed acutely in specific services; something more multidisciplinary and multi‐professional is needed (Int. 4 ED).

A psychiatric emergency department can certainly address urgent needs, but the problem is that it often involves many related issues of a medical nature. Integration between different disciplines is essential (Ιnt. 22 ΕD).

…and organisational‐management reasons.

The emergency department is often congested, and management in the ED is not easy. Neither in terms of time nor resources (Int. 17 ED).

Even the most saintly nurse, faced with 200 admissions a day, flips out when dealing with the requests of psychiatric patients (maybe an euro or a cigarette) (I5 MH).

### 4.2 Separated Accesses: Stigma?

All mental health professionals agree that direct access is a strong element of stigma:

It's like saying you have preferential access because you're made that way, you're psychiatric! (Int. 9 MH).

It increases in stigma among the population and among doctors of all categories, including psychiatrists who are not exempt from stigma; it risks excluding, separating, and offering lower quality service. Threat to the patient's right to health (I2 MH).

Then a nurse clinical manager of an acute unit:

First evaluate me like everyone else, then treat me based on the pathology, even if I am ugly, dirty, and bad! (I5 MH).

Stigma seems to be present among emergency personnel. This impression is also confirmed by an ambulance nurse from the emergency department.

I see stigma in the ED when I bring these patients. They are seen as a hassle and a waste of time. (I8 ED).

But also:

Those who are not known patients go to the Emergency Department, while the known ones want to go directly to psychiatry, so they are happier. (I1 ED).

But there is an interesting point for reflection:

A patient who is directed to a Psychiatric ED is inevitably ‘branded’ according to social conventions; this can represent a heavy label for those who access it. It is equally important to understand that each pathology has a very specific destination structure for treatment, so despite the possible negative connotation associated with the Psychiatric ED, it is essential to go beyond the preconception in light of the real need for this service (I14 ED).

A member of SIMEU give a similar explanation

Each discipline would benefit from a dedicated pathway, similar to Fast Track (I21 ED).

But:

It might almost seem like a return to the old asylums. It's like a label, it's a totalitarian structure. Psychiatric patients should be treated like everyone else, with standard treatment, even in the general ED. The fast track would be better than direct access; I would say the balance always leans towards the general ED rather than the specialized ED (I4 ED).

### 4.3 ED Staff: Trained to Manage Patients With Psychiatric Disorders?

#### 4.3.1 MH Staff: Trained to Receive Emergency Patients?

The final families concern whether ED personnel are prepared to manage an urgent patient with psychiatric manifestations and, conversely, whether MH staff are capable of managing the somatic emergencies of psychiatric patients.

We are a bit ‘rusty’ on organic issues and might have a partial observation of signs and symptoms (I16 MH).

Emergency Department staff inherit a medical culture that currently does not dedicate much time to relationships and psychiatric issues (I8 MH).

We can already notice how the proposed solutions involve training, spaces, and multidisciplinarity.

Mental health professionals are not very accustomed to organic problems; they should meet with emergency‐urgent care personnel, undergo updates and also training (I22 MH).

The suggestion is to evaluate the competency profile of the ED nurse and understand what is lacking for psychiatric patients, and for those in MH, the same thing applies to the critical patient aspect (I4 ED).

Even the most saintly nurse, faced with 200 admissions a day, flips out when dealing with the requests of psychiatric patients (maybe an euro or a cigarette); we need to define interventions for staff and spaces to ensure everyone is treated optimally. Providing answers to everyone who accesses the service, even if it is utopian! (I5 MH).

## 5 Discussion

The literature has already extensively addressed the issue of psychiatric patients in the ED, but previous studies have compared the perceptions among ED members (Fleury et al. 2024; McIntosh 2021) or those of patients (McIntyre et al. 2024), but not between professionals of different services on how to support a patient who ‘belongs’ to mental health but frequently uses ED services. This research perspective seems interesting because the interviews reveal mutual prejudices. For example, one interviewee (I1ED) mentioned that psychiatrists are perceived to arrive whenever they feel like it, while another interviewee (I7MH) expressed the belief that those who want direct access to the ED are asylum advocates and should be labelled as such. These prejudices and misunderstandings about each other's professionalism create obstacles in the organisational mechanisms of service integration and, above all, in patient care. Among the main findings of the study, it was noted that the primary concern of mental health professionals is that a dedicated emergency department could lead to patient stigmatisation. This phenomenon is not new and is also present among mental health professionals themselves affecting the quality of care (Sreeram et al. 2022). These prejudices are already described in the literature by the patients, as shown in the studies by McIntyre et al. (2024) and Bull et al. (2024), as well as by ED nurses (Keslar 2024; McIntosh 2021).

Such prejudiced attitudes seem to be confirmed by some of our interviewees. One interviewee (I1ED) described the patient as happier to go directly to psychiatry, while another (I12 ED) expressed frustration, noting that the psychiatrist arrives and they must handle the situation in the meantime. Additionally, another interviewee (I8 ED) mentioned seeing stigma in the ER when bringing these patients.

In short, it seems plausible that, in EDs dramatically dealing with the phenomenon of overcrowding (Pearce et al. 2024) as recognised by many interviewees from both areas, the psychiatric patient is not seen as a real patient deserving of care but rather as something to be treated quickly and sent for consultation (Grace 2020). Healthcare facilities have been aware of this problem for decades (Wand and Happell 2001; Tucci et al. 2017), and over these decades, many alternative systems for managing the access of psychiatric patients, different from general ED access, have been experimented with. All these models seem to be effective in managing psychiatric emergencies and in providing respectful and psychosocial care for patients (Postorivo et al. 2024; Anderson et al. 2022; Schmidt et al. 2020), but there is no clear data in the literature regarding their effectiveness in managing physical emergencies.

Therefore another finding is that the main concern of the interviewed mental health staff is that, even with these differentiated pathways, patients might not receive adequate differential diagnoses. This means that physical symptoms could be underestimated in favour of focusing on behavioural and psychiatric symptoms, leading to diagnostic overshadowing, which is well described in the literature (Molloy et al. 2023; Tucci et al. 2017; Mather et al. 2014). As some interviewees state, the background of emergency department staff, both doctors and nurses, is medical and organic in nature, whereas that of mental health staff is more focused on the patient and their social functioning (Rapisarda and Miglioretti 2019) even in an emergency setting (Schmidt et al. 2020).

In response to this problem, the solution, according to the MH cohort of this study, is fast‐track pathways, with an initial differential evaluation in the general ED and then care by MH staff. On this point, the two cohorts seem to converge, even if triage nurses would see the fast‐track pathway, if not direct access, for known patients, while for unknown patients, a complete medical evaluation followed by possible transfer.

This might suggest that many patients, in the perception of triage nurses, are ‘frequent users’, low‐priority white codes, who would deserve a community response rather than ‘clogging up’ the ED. This is also described in the literature: indeed, according to the systematic review by Sacre et al. (2022), being known has an impact on the quality of care provided, not always positive, and often known patients are considered responsible for overcrowding. However, they are less feared than unknown patients, who are considered potentially dangerous (McIntosh 2021).

But the concern expressed by MH has a strong ethical and cultural basis among our cohort, both psychiatrists and nurses, who see the adoption of dedicated pathways as a violation of the patient's citizenship rights, a form of stigmatisation, and a return to the separation between the ‘sane’ and the ‘insane’; a place as described by Schmidt et al. (2020) where dedicated psychiatric facilities guard and protect, taking care of the person's suffering, in contrast with what Postorivo et al. (2025) report, which describes their ED experiences as characterised by feelings of insecurity, loneliness, intimidation, and stigma. In the face of some opposition between the two cohorts, the solution proposed by the interviewees, particularly the nurses, is organisational integration starting with training through dedicated courses. Indeed, in an almost mirror‐like manner, both cohorts admit to having training needs to manage an emergency involving a psychiatric patient. MH nurses admit to being ‘rusty’ (I16 MH) in organic assessment, especially in emergency situations. This assertion is supported by Dickens et al. (2019) for MH nurses. Conversely, the issue of limited preparation for psychiatric symptoms is also described in the literature (Keslar 2024; Mcintosh 2023) for ED staff. Specifically, the courses proposed by the interviewees include specific courses for the ED (Advanced psychiatric and mental health training, relational and patient approach, management of aggressive‐agitated patients) and for MH (courses based on the integration of ED skills, enhancement of management for organic pathologies).

However, it remains uncertain how much formal training challenges ED nurses and how much a complex multiplicity of factors (McIntosh 2023) is involved. Additionally, meetings between ED and MH in a multiprofessional perspective, as well as strengthening the liaison between services through the intervention of MH nursing staff from both the hospital ward and the community, could be beneficial. It appears that the two cohorts suggest a solution that considers the needs of both professional groups. This involves greater integration between departments at both the macro level, among management, and the micro level, among clinical staff. The creation of fast‐track pathways that do not exclude a thorough evaluation of potential organic aspects upstream is essential. This organisational structure must be supported by well‐prepared and well‐trained clinical personnel.

The limitations of the study naturally include the inability to generalise the results. Our findings derive from a purposeful sample and represent a specific clinical reality with its own distinct peculiarities. We decided to gather the opinions and perceptions of professionals from a descriptive perspective, attempting not to pass judgement but to best represent what we believe are expressions of professional cultures, both worthy of respect but also of analysis, as they influence the quality of care.

## 6 Conclusions

To our knowledge, this is the first study that compares the opinions of two different professional points of view, two professional cultures, on this specific topic: the treatment of psychiatric patients in the ED. In our study, it appears that the two cohorts are immersed in their respective professional cultures. This is evident from the content brought up in the interviews, but even more so from the style and terminology used by the different participants. Professional cultures view the same problem through different sensitivities and perspectives that, in this study, do not always align, proposing different clinical and organisational solutions. Significantly, despite this apparent opposition, all interviewees are open to dialogue and professional exchange as a first solution to the issue of psychiatric patients in the ED.

We believe that some, if not many, of the opinions observed can also be found among professionals in other national contexts beyond Italy. Nonetheless, the insights presented in this study can serve as a stimulus for reflection.

Within this debate, the patient with mental disorders, the Person, should not be left in the background. Too often, they find themselves balancing between nervous and increasingly crowded EDs with equally nervous users, and MH services torn between ethical demands and organisational‐pragmatic care approaches that are not always intimately well accepted.

The patient must remain central and should be the pivot around which all clinical and organisational solutions presented in this study are organised, taking into account their healthcare, care, and social rights equally.

## 7 Relevance for Clinical Practice

Nurses are on the front line and have significant responsibilities for people with psychiatric disorders who come to the ED. The competence to welcome, assess, and then manage these patients has considerable clinical, organisational, and ethical implications that are often underestimated or misinterpreted by professionals. We believe that our study can help in self‐reflection on one's own practice and understanding of others'.

## Ethics Statement

The authors have nothing to report.

## Consent

The authors have nothing to report.

## Conflicts of Interest

The authors declare no conflicts of interest.

## Data Availability

The data that support the findings of this study are available from the corresponding author upon reasonable request.
